# Extracellular Vesicles: The Future of Diagnosis in Solid Organ Transplantation?

**DOI:** 10.3390/ijms24065102

**Published:** 2023-03-07

**Authors:** Nekane Romero-García, Javier Huete-Acevedo, Cristina Mas-Bargues, Jorge Sanz-Ros, Mar Dromant, Rafael Badenes, Consuelo Borrás

**Affiliations:** 1 Freshage Research Group, Department of Physiology, Faculty of Medicine, University of Valencia, 46010 Valencia, Spain; 2 Centro de Investigación Biomédica en Red Fragilidad y Envejecimiento Saludable, Instituto de Salud Carlos III (CIBERFES, ISCIII), 28029 Madrid, Spain; 3 Instituto Sanitario de Investigación INCLIVA, 46010 Valencia, Spain; 4 Department of Anesthesiology and Surgical Trauma Intensive Care, Hospital Clinic Universitari de Valencia, University of Valencia, 46010 Valencia, Spain; 5 Department of Cardiology, Hospital Universitari i Politècnic La Fe, 46026 Valencia, Spain

**Keywords:** transplant, exosomes, diagnostic

## Abstract

Solid organ transplantation (SOT) is a life-saving treatment for end-stage organ failure, but it comes with several challenges, the most important of which is the existing gap between the need for transplants and organ availability. One of the main concerns in this regard is the lack of accurate non-invasive biomarkers to monitor the status of a transplanted organ. Extracellular vesicles (EVs) have recently emerged as a promising source of biomarkers for various diseases. In the context of SOT, EVs have been shown to be involved in the communication between donor and recipient cells and may carry valuable information about the function of an allograft. This has led to an increasing interest in exploring the use of EVs for the preoperative assessment of organs, early postoperative monitoring of graft function, or the diagnosis of rejection, infection, ischemia-reperfusion injury, or drug toxicity. In this review, we summarize recent evidence on the use of EVs as biomarkers for these conditions and discuss their applicability in the clinical setting.

## 1. The Interest of EVs in Solid Organ Transplantation

### 1.1. The Thriving Field of Solid Organ Transplantation

Solid organ transplantation (SOT) has developed from an experimental treatment in the 20th century to the standard of care for patients suffering from end-stage organ failure [1]. In 2021, 144,302 solid organs were transplanted in the European Union (EU) according to the Spanish National Transplant Organization, which represents a 19.1% increase from 2010 [2]. The exponential growth in the elderly population over the last decades, which requires cost-effective solutions to non-communicable diseases, plays a role in the lengthening of the transplantation waitlist [3]. The kidney is the most frequently transplanted organ and is the gold standard for renal replacement therapy, which provides better survival and quality of life than dialysis [4]. It is followed by the liver and heart, which are transplanted as the last resort in organ failure. Lungs, pancreas, pancreas-kidney, and intestine transplants are common practices today; more novel transplants, such as cornea, pancreatic islet, or liver fraction transplants are still being implemented in major hospitals [5]. Improvements in surgical techniques have led to more successful multi-organ transplants with fewer complications and reduced systemic injury. Additionally, immunosuppression therapy has been refined to minimize the host’s immune response and improve the survival rate of transplanted organs. All these improvements have resulted in an impressive survival benefit for patients, estimated as 2,270,859 life-years saved globally during the last 25 years, and a mean of 4.3 life-years per transplant recipient [6].

Despite these advances, SOT is currently facing a major challenge: the shortage of organ availability, which is outpaced by the steadily growing need for transplants. By the end of 2019, more than 58,000 patients remained on the waitlist for a transplant, with an associated mortality of 3–4% while on the waitlist [2]. Due to the recent stagnation in transplant metrics since 2017 in the EU, to enhance transplant activity, a roadmap has recently been proposed by experts [7]. Suggested measures include (i) optimizing less frequent types of donations, such as living donation (LD), donation after circulatory death (DCD), or xenotransplantation in preclinical studies, and (ii) promoting long-term graft survival. Interestingly, the increase in short-term graft survival achieved in the last decades has not been matched by a similar increase in the long term, as pointed out by studies on different organs [8,9,10]. This phenomenon can be attributed to insufficient resources for longitudinal monitoring and a deeper understanding of the biological processes after transplantation. Causes involved in the long-term loss of organ function include rejection and infections, whose early diagnosis is complex and requires invasive techniques. In the search for both adequate donors and a thorough assessment of graft function, the European Kidney Health Alliance has strongly encouraged research into novel SOT-specific biomarkers [11].

### 1.2. Intercellular Communication through Extracellular Vesicles

Intercellular communication is a critical function of multicellular organisms. While its integrity is fundamental to homeostasis and health, its impairment lies at the root of conditions such as aging or cancer [12]. The role of intercellular communication during SOT is mainly studied from the perspective of the immune response. For instance, the recipient’s antibodies or inflammatory cytokines can help identify an altered response of the recipient to the graft [13]. However, the immune system is only a small piece of the bigger puzzle of intercellular communication between the graft and the host. In general, intercellular communication can be mediated by three pathways: direct contact through membrane receptors, secretion of soluble mediators, and release of extracellular vesicles. Soluble particles, such as the aforementioned antibodies or cytokines, have traditionally been used as biomarkers due to their availability; however, they present some disadvantages, such as their short half-lives, their susceptibility to degradation by extracellular enzymes, and the impossibility to track down their tissular origin. In the last decades, communication through extracellular vesicles (EVs) has gained attention, thanks to its potential to meet these shortcomings [14]. In the context of solid organ transplantation, EVs have been shown to be involved in the communication between donor and recipient cells and may carry information about the state of the transplanted organ. This has led to an increasing interest in exploring the use of EVs as diagnostic biomarkers in SOT [15].

**Figure 1:**
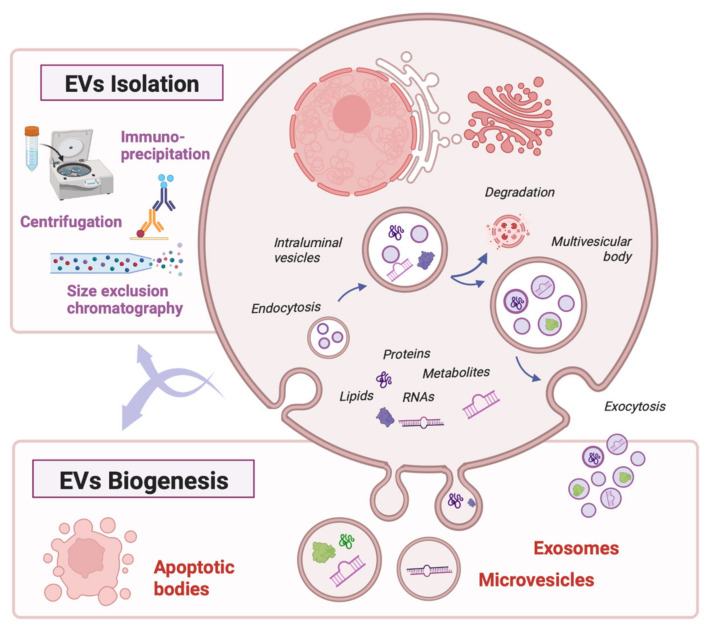
EV biogenesis, cargo, and isolation methods. Created with biorender.com, accessed on 26 February 2023.

Extracellular vesicles (EVs) are key mediators of intercellular communication. According to the latest consensus, they can be defined as those particles naturally released by cells, which are enclosed by a lipid bilayer and do not contain a functional nucleus. They vary in size from 50 nm to up to 2000 nm in diameter, and they are accordingly classified as small EVs (those under 200 nm) and medium or large EVs [16]. EVs can originate through different biogenetic pathways inside the parent cell, splitting into microvesicles, exosomes, or apoptotic bodies [17] (Figure 1). According to the distance they travel, they can function as autocrine, paracrine, or endocrine mediators, and they interact with the target cell either through membrane receptors or via endocytosis [18]. All families of biomolecules are represented in their cargo: proteins, lipids, metabolites, and nucleic acids [19]. Among them, some compounds are particularly relevant in the field of organ transplantation, such as active enzymes, membrane receptors, cytokines, mRNA transcripts, or miRNAs. Notably, this cargo is dependent on the type of parent cell and can vary widely from physiological to pathological conditions, reflecting their metabolic state and rendering them as potential biomarkers [20]. On the other hand, it has also been shown that cells have the capacity to selectively load some molecules into EVs, such as miRNAs found only in low concentrations in the cytoplasm, to modulate gene expression in distant cells [21].

The growing interest in EVs is partly due to the wide range of physiological functions they are involved in, including the immune response [22,23], tissue remodeling and repair [24,25,26], stem cell pluripotency [27,28], angiogenesis [29,30], and coagulation [31]. The immune response was one of the first functions discovered when Raposo et al. showed that B cells secreted EVs to present antigens to T cells [32]. Other studies have shown that dendritic cells take up circulating EVs from other dendritic cells, and their cargo proteins are processed and presented as antigens, playing a role in immune regulation [33,34]. Among other pathological conditions they are involved in, cancer has received the most attention [35,36], but EVs also play a role in neurodegenerative diseases such as Alzheimer’s [37] and cardiovascular diseases such as atherosclerosis [38], as well as infectious diseases such as HIV-1 infection [39].

### 1.3. EVs as Stable, Organ-Specific Biomarkers of Health and Disease

The potential of EVs as biomarkers in end-stage organ failure has been widely explored in recent years. Compared with soluble biomarkers, EVs provide the advantages of high stability in the extracellular medium, longer half-lives, and information about their parent and target cells [40]. In kidney diseases, EVs have been studied in the diagnosis of acute kidney injury, chronic kidney disease, renal transplantation, thrombotic microangiopathies, vasculitis, IgA nephropathy, nephrotic syndrome, urinary tract infection, cystic kidney disease, and tubulopathies [41,42,43,44]. They are also useful in diagnosing and grading the prognosis of heart failure [45,46]. Nonetheless, some of these conditions have been widely studied for many years; therefore, several soluble biomarkers already exist that are well integrated into clinical practice, as is the case for brain natriuretic peptides (BNP and NT-proBNP) in heart failure. In contrast, the utility of these biomarkers in graft function monitoring is not fully established and requires further study [47]. Regarding BNP, it has been found that it tends to remain high after transplantation, even with no evidence of left ventricular function [48]. Thus, the need for novel biomarkers to monitor the function and detect potential conditions affecting the integrity of an allograft has drawn attention to the field of EVs.

EVs from different cell types are present in nearly every body fluid, from plasma to synovial fluid, including breast milk, saliva, and urine [49]. In the field of organ transplantation, most studies use EVs from plasma, urine, or perfusion fluid, given their availability in the volumes needed for most isolation protocols [50,51]. Isolation methods include ultrafiltration, size exclusion chromatography, and immunoaffinity-based techniques [52,53], although most studies in SOT use ultracentrifugation since it is a cost-effective technique that reaches high purity rates [51,54] (Figure 1). An additional benefit of EVs compared with soluble biomarkers is the possibility to discern whether they come from the donor or the recipient, shedding light on the underlying immune pathways at the time of the transplant. Donors’ and recipients’ EVs are most frequently identified through imaging flow cytometry based on the staining of specific mismatching HLA complexes [55].

## 2. EVs as Diagnostic Tools in Solid Organ Transplantation

### 2.1. Search Strategy

**Figure 2:**
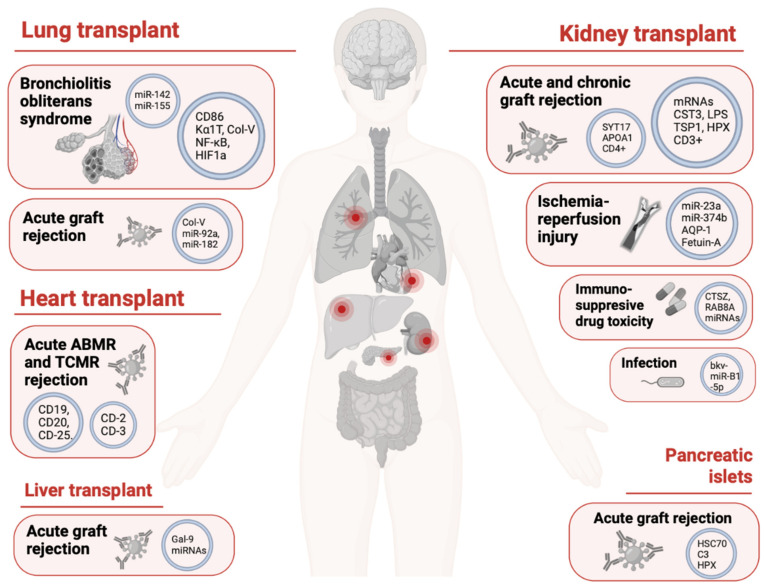
EVs as biomarkers in solid organ transplantation. The main EV-contained biomolecules involved in the diagnosis of the different conditions specific to each organ are displayed. ABMR: antibody-mediated rejection; TCMR: T-cell-mediated rejection. Created with biorender.com, accessed 3 January 2023.

The current narrative review aims at summarizing the current knowledge on the use of extracellular-vesicle-derived components as biomarkers in a range of conditions associated with SOT. Original research studies were identified by searching the Medline (PubMed), Embase, Web of Science, and Google Scholar databases from their inception. The main search was run on 20 December 2022 and updated on 3 January 2023. The keywords ‘solid organ transplantation’ (transplant, graft, kidney transplant, liver transplant, lung transplant, heart transplant, intestine transplant, pancreatic islets transplant, or corneal transplant), ‘extracellular vesicles’ (exosomes, exosomal, or microvesicles), and ‘diagnosis’ (complications, rejection, allograft rejection, acute rejection, chronic rejection, infection, drug toxicity, graft function, graft quality, or ischemia-reperfusion injury), or any of their synonyms listed in brackets, were typed in various combinations using Boolean operators. Queries were limited to those studies involving mammalian subjects and an in vivo design, with full texts available. Hand searches of the reference lists of articles and relevant literature reviews were used to complement the computer search. The search focused solely on articles in English published in peer-reviewed journals to enhance the methodological rigor. Previous reviews, position papers, and case reports or case series were excluded. Figure 2 summarizes some of the most relevant findings.

### 2.2. Preoperative and Postoperative Assessment of Donor Organ Function

Assessing the function of donor organs non-invasively at the time of transplantation is a crucial goal to increase graft survival rates, as well as to ensure donor safety in living donation. Traditionally, assessment of organ function has relied on laboratory parameters, such as glomerular filtration rates for kidneys, and imaging techniques. Some studies propose that the accuracy of the glomerular filtration rate, especially with near-normal kidney function, may be suboptimal; for this reason, new soluble biomarkers, such as cystatin C, are gaining importance [56]. This goal gains relevance as the number of organs from deceased donors (DDs), particularly DCDs, increases. Many factors explain the poorer outcomes of organs transplanted from DDs versus LDs, such as the younger donor age or the planned surgery, and the less strict screenings of graft function may also play a role. Moreover, pretransplant evaluation relies mostly on preexisting medical conditions and biopsies, which are not exempt from risk [57].

EVs provide different advantages in the preoperative evaluation of donor organs, as summarized by Ashcroft et al. [50]. In a kidney transplant, a study by Turco et al. found that specific populations of urinary EVs (uEVs) can indicate aging-related structural changes in living donor kidneys. Both the number of EVs and their cellular origin changed with conditions such as nephrosclerosis or nephron hypertrophy [58]. Another study by Lozano-Ramos et al. also found that uEVs can be used to assess donor kidney function by analyzing their miRNA profile. They compared EVs from LDs and DDs and found no overall differences in miRNA profiles in normofunctioning grafts at one year. Interestingly, only miR-326, which targets the pro-apoptotic protein Bcl-2, was overexpressed in living donors [59]. Notably, EVs have also been isolated from the preservation fluid of organs both in DCDs and brain death donors (DBDs). These EVs, secreted by the renal endothelium, contain miRNAs that might be able to predict early or delayed graft function (DGF) [60].

EVs have also shown a role in the early assessment of postoperative graft function. Regarding kidney transplantation, research has been conducted to identify specific patterns of EVs in urine or blood related to DGF. DGF is defined as acute kidney injury that occurs in the first week of kidney transplantation, necessitates dialysis intervention, and is associated with higher rates of acute rejection and shorter graft survival in the long term [61]. Some recent studies have found specific EV components with high prediction accuracy for DGF, as is the case for CD133 as an EV membrane marker [62], neutrophil gelatinase-associated lipocalin (NGAL) [63,64], and individual miRNAs [65]. Other works provide a more global picture through whole proteome analysis [66] or EV-contained miRNA panels [67]. Differential diagnosis of acute graft dysfunction is another current challenge that could be addressed through EVs. Currently, a combination of laboratory tests (e.g., GFR and proteinuria), immunological findings (e.g., donor-specific antibodies), imaging techniques (e.g., Doppler ultrasound), and histological parameters is needed to differentiate between conditions such as rejection, infection, drug-induced damage, ischemic injury, recurrence of the primary disease, or surgery-related vascular or urinary tract complications. Matignon et al. proposed an mRNA signature in urinary cells to successfully differentiate some of these conditions, reducing the number of biopsies in these patients [68]; a similar uEV-based approach may be of use to this end.

### 2.3. Diagnosis of Graft Rejection

Despite the recent technical advances and better outcomes achieved, graft rejection remains the Achilles’ heel of SOT [69,70,71]. Graft rejection can be defined as the loss of allograft graft function caused by the recipient’s immune system. Acute rejection (AR) occurs within the first few weeks or months after transplantation and is caused by a rapid and strong immune response to the transplanted tissue [13]. This type of rejection is usually prevented and treated with immunosuppressive drugs [72]. However, even short episodes of graft rejection can have long-term consequences on a liver graft, including an increased risk of failure and mortality. Chronic rejection, which occurs after the first year post-transplant, is less common and responds poorly to treatment, leading to permanent organ damage [13]. Depending on the immunological mechanisms, rejection can be divided into antibody-mediated (ABMR) or T-cell-mediated (TCMR) rejection, with different treatment strategies and outcomes [69].

Hence, there is an urgent need for improved methods for immune response monitoring transplant recipients. Despite all available laboratory parameters and imaging techniques, histological examination remains the gold standard for the diagnosis of rejection. Thus, serial surveillance biopsies are the standard of care in heart and lung transplantation to enable early therapeutic intervention; kidney biopsies may also be needed if there is a diagnostic concern. Nonetheless, biopsies are associated with a risk of bleeding and damage to the allograft or the surrounding organs [73]. Regarding pancreatic islets, neither biopsy nor imaging is available; therefore, islet function is monitored mostly through c-peptide concentrations and glycemia [74]. Therefore, the development of non-invasive biomarkers to detect immune-mediated allograft injury is required for clinicians to tailor immunosuppression and intervene early, ideally before any visible organ dysfunction occurs [15].

Kidney graft rejection is the main cause of graft failure censored for death at any time following transplantation [75]. According to some series, the incidence of ABMR increases over time at a rate of 1.1% per year, while TCMR is rare 6 years after transplantation [75]. The differential diagnosis of graft rejection in kidney transplants is an ongoing challenge, since other conditions, such as drug toxicity or infections, may simulate rejection, particularly in the long term. The current diagnosis of chronic allograft failure through serial biopsies poses a problem since, aside from the well-known risks of bleeding and infection, the percentage of inconclusive samples is considerable [76]. Most authors study uEVs in the quest for biomarkers of AR. Proteomics analysis has provided several candidates, such as cystatin C (CST3), lipopolysaccharide-binding protein (LBP) [77], tetraspanin 1 (TSPAN1), and hemopexin (HPX) [78]. Some studies have compared these proteins to soluble urine biomarkers and identified some that are specific to EVs [79]. An mRNA panel has been shown to outperform laboratory kidney-function-based methods in early diagnosis or AR while still being able to differentiate between their immune mechanisms [80]. Urinary EVs from T cells are also useful since an increase in membrane marker CD3 has shown specificity to TCMR [81]. Studies on plasma-derived EVs have identified some EV subpopulations linked to AR, which can also be used to monitor responses to treatment [82]. Others have focused on mRNAs and have found a combination of four genes that can accurately predict ABMR [83].

As for chronic kidney rejection, several EV-based biomarkers are currently under study. While some studies can identify this condition based on a single biomarker in uEVs [84], others have proposed a combination of proteins to this same end [85]. Interestingly, uEVs of renal origin can differentiate chronic rejection from other confounding conditions, such as calcineurin inhibitor toxicity, in which biopsies and laboratory assays are frequently needed [84]. Membrane markers in EVs with an immune origin, such as T helper cells, can also shed light on the underlying cause of graft failure, as shown by Yang et al. [86].

Liver and pancreas rejection studies mostly make use of plasma-derived EVs. In models of liver rejection, protein galectin-9 revealed an accurate diagnosis of TCMR, and several miRNAs were found to be over- (miR-223 and let-7e-5p) and under-expressed (miR-199a-3p) in TCMR [87]. The only study performed in this field is based on a human-into-mouse xenogeneic islet transplant model of pancreatic islets. The authors found that mice with AR showed a decrease in donor EVs and an increase in T-cell EVs from the recipient. The potential of donor EVs as biomarkers of rejection has previously received attention in a kidney transplant model, where CD9+ HLA-A3+ EVs from the donor increased only in recipients with no allograft dysfunction [88]. Furthermore, they found four proteins that were overexpressed in mice with induced AR compared with controls. The clinical interest of these findings is reinforced by the fact that these biomarkers precede classic manifestations of organ dysfunction, such as hyperglycemia [89].

The incidence of heart transplant rejection has steadily dropped in recent years, from 30.5% in 2004–2006 to 24.10% in 2010–2015, from discharge to 1 year of follow-up [90]. Nonetheless, they remain among the highest rejection rates in SOT. The diagnosis of rejection relies on endomyocardial biopsy (EMB) as well as donor-specific antibodies. In addition to usual risks, EMB increases the risk of tricuspid regurgitation [91]. Studies have found candidate soluble biomarkers in plasma, such as microRNA, mRNA profiling, and detection of circulating cell-free DNA; however, these are only stable for a short time in plasma [92]. For this reason, EVs have emerged as tools for rejection monitoring. Preclinical studies in mice have shown that simple measures, such as total EV concentrations in plasma, can accurately predict heart AR at an early stage, at which biopsies still show insignificant or grade 0R changes [93]. In humans, EV-based models have been able to diagnose AR and its two immunological variants, ABMR and TCMR, with adequate sensitivity and specificity. Castellani et al. based their model on mostly membrane proteins [94], while Kennel et al. performed a proteomic analysis [95].

In lung transplants, recent studies have succeeded at early diagnosing both acute lung rejection and the most common manifestation of chronic rejection, bronchiolitis obliterans syndrome (BOS). BOS, a chronic obstructive pulmonary disease (COPD)-like clinical pattern, affects about 50% of transplanted patients within 5 years [96,97] and accounts for more than 30% of the mortality rate after this period [97]. Early diagnosis and treatment of AR can prevent it from evolving into chronic rejection; however, diagnosis relies on a CT scan and lung biopsy, which have limited sensitivity [98]. Hence, intense surveillance for AR is limited, reducing early recognition. Some recent studies have investigated the use of EVs in bronchoalveolar lavage fluid (BALF) to generate a molecular fingerprint of AR. Gregson A. et al. performed an mRNA analysis and found a transcriptomic signature that accurately characterized patients with AR [99]. Another work by Gunasekaran et al. analyzed proteins and miRNAs in both plasma-derived EVs and BALF EVs in healthy recipients and compared them with those of lung transplant patients with AR or BOS. They found that donor HLA molecules and lung-associated self-antigens, such as collagen-V (Col-V) and K alpha 1 tubulin (Kα1T), were overexpressed in both conditions and could lead to an earlier diagnosis by up to 6 months. Several EV-contained miRNAs related to inflammation and endothelial activation, as well as to the expression of certain costimulatory molecules, could accurately identify these conditions [100]. In a more recent study by the same group, plasma-derived EVs from BOS patients were isolated, and their proteins and transcription factors were analyzed, further expanding the candidate biomarkers for BOS diagnosis. Moreover, when healthy mice were treated with the aforementioned EVs, they developed a proinflammatory phenotype consisting of antibodies against self-antigens and increased IL-17 and IFN-γ, and decreased IL-10. Thus, they suggest that EVs produced during rejection have immune-boosting qualities and play a significant part in chronic rejection after lung transplantation.

In this line, some studies have also focused on the role of native EVs in the pathophysiology of rejection [22,54]. It is now known that allograft recognition does not always occur through the direct recognition of donor cells. Instead, the immune response leading to graft rejection can be triggered by EVs carrying donor MHC molecules and peptides. Studies have shown that host antigen-presenting cells (APCs) in lymph nodes can present EVs bearing donor MHC I and II molecules, which initiates T cell activation after skin and heart transplants [101]. This suggests that host APCs can acquire donor MHC molecules present on EVs secreted by donor cells, and, hence, EVs would be responsible for determining the fate of the allograft through a semi-direct pathway. Other studies have described the role of EVs in allograft recognition through an indirect pathway, whereby the EV-presented antigen is taken up and processed by B lymphocytes before being presented to the T cell [102].

### 2.4. Diagnosis of Ischemia-Reperfusion Injury

Ischemia-reperfusion injury (IRI) is a condition affecting most transplanted organs, particularly when they are derived from donations after circulatory death (DCDs), due to the longer times of warm ischemia. However, it is also present in most organs susceptible to ischemia of any cause, such as myocardial infarction. The pathophysiology of IRI is complex: while the imbalance between metabolic supply and demand causes tissue hypoxia and microvascular dysfunction, subsequent reperfusion boosts innate and adaptive immune responses and activates the cell death machinery [103]. EVs garner interest for the differential diagnosis of IRI versus other causes of DGF in the postoperative setting. Sonoda et al. propose aquaporin 1 (AQP1) as an early negative biomarker of IRI; according to their study, the decrease in AQP1 in uEVs may be a consequence of both decreased release and production and may be useful for diagnosing IRI within the first 6 h, before changes in renal function parameters are observed [104]. Nonetheless, other studies propose AQP1 reduction as a constant phenomenon in kidney transplantation [105]. Some of these biomarkers stand as potential targets to minimize IRI-related damage; for instance, Li et al. found that EV-contained miR-23a, which increases in IRI in response to hypoxia-inducible factor 1, could be targeted to limit inflammation of the renal parenchyma [106]. Similar results were obtained with miR-374b-5p [107].

### 2.5. Diagnosis of Immunosuppressive Drug Toxicity and Graft Infection

Immunosuppressive drugs are responsible for the remarkable increase in graft survival during the last decades [72]. Despite their long-known side effects, such as nephrotoxicity, calcineurin inhibitors (CNIs) remain the cornerstone of immunosuppression in kidney transplantation. Chronic CNI toxicity (CNIT) can result in vascular dysfunction, interstitial fibrosis, and tubular atrophy, compromising the integrity of the graft [108]. Many factors account for nephrotoxicity, the most important of which is drug dosing; however, a non-negligible interindividual variability exists, since side effects have been reported even with low doses. For this reason, drug levels in plasma and serial biopsies are losing ground in favor of non-invasive strategies for pharmacokinetic monitoring. Proteomic and miRNA analysis of urinary EVs in kidney transplantation have shed some light on the question, according to some recent studies. Carreras-Planella et al. identified members of the uroplakin family as predictors of CNIT over healthy and of-other-cause kidney fibrosis [109]. Costa da Freitas et al. used a similar approach to correlate uEV-contained miRNAs with tacrolimus levels [110]. This is in line with previous studies demonstrating the potential of EVs to monitor immunosuppressive treatment in autoimmune diseases [111].

Post-transplant infection is one of the most feared complications, given the high morbimortality it accounts for both in the short and the long term [112]. Early diagnosis of infection may be delayed by the atypical clinical manifestations of transplanted patients under immunosuppressive regimes. Moreover, infection screening through laboratory parameters generally requires biopsy confirmation, as is the case for BK polyomavirus (BKV) in kidney transplant recipients [113]. Although its incidence has dropped in the last decades, BKV is still a prevalent cause of nephropathy, affecting up to 10% of kidney recipients and causing allograft failure in 10 to 80% of these [114]. Hence, novel biomarkers of infection in SOT are currently under development, which could help to initiate prompt treatment and achieve adequate balance in immunosuppressive therapies [115]. Kim et al. proved that, aside from human miRNAs, viral miRNAs (miR-B1-5p and miR-B1-3p) could also be used as biomarkers of infection with high sensitivity and specificity [116]. These findings were supported by a previous study on kidney biopsies, wherein the same viral miRNAs were found [117]. In lung transplants, the potential of EVs goes beyond that of a diagnostic tool; they also represent the mechanism through which infections relate to long-term graft dysfunction and rejection, as proven by Gunasekaran et al. [118] (Table 1).

## 3. Opportunities and Future Directions

The use of EVs as diagnostic tools in SOT is a rapidly growing field of research. Evidence suggests that EVs can provide valuable information about the function of transplanted organs, allowing for early detection of complications such as rejection or infection. As research progresses, EVs are likely to become widespread biomarkers, providing important benefits for patients and physicians alike. However, at least three challenges must be addressed before they are fully implemented in the clinical setting. First, standardizing EV isolation and characterization procedures is necessary to generate homogeneous research that can be compared and meta-analyzed. This is particularly applicable to urinary EVs, as highlighted by the ISEV. Most studies on kidney transplants use uEVs, since they are easily available and non-invasive, and urine is already routinely collected to measure renal function parameters. However, current investigations on uEVs should address certain biases, such as the variable uEV concentrations or the wide range of isolation methods available, which affects the reproducibility of the studies. As possible solutions, the normalization of uEV concentrations to urine dilution and the use of flow cytometry to identify specific uEV populations have been proposed [122]. Additionally, it is important to move from the study of single biomarkers to that of full diagnostic panels, which are cost-effective and feasible for clinical use. Thus, there is a manifest need for clinical studies that validate the use of EVs as efficient biomarkers in SOT, through their comparison with traditional biomarkers or diagnostic criteria. This is aligned with the 2018 insight paper from ISEV, which remarks on the need to evolve from basic to applied research that takes full advantage of the potential of EVs. Finally, future studies should aim not only at diagnosing a certain condition but also at solving frequent issues of clinical practice. For instance, instead of looking for biomarkers of rejection, future studies should, rather, look for biomarkers that establish the differential diagnosis of graft dysfunction, and therefore help decision-making. Other clinical situations where EVs could be of help are in the monitoring of responses to immunosuppressive or antimicrobial therapy. In general, a thorough study design to include control patients who resemble those in the clinical setting would be key to this goal. Addressing these challenges is crucial for ensuring that extracellular vesicles realize their full potential as a diagnostic tool in solid organ transplantation.

## Figures and Tables

**Table 1:** EVs as diagnostic resources in solid organ transplantation.

Research Topic	Organ	Study Population	EVs Origin	EVs Component	Reported Outcomes	Reference
Preoperative donor organ assessment	Kidney	LD	Urinary EVs	Various membrane protein signatures	Kidneys with nephrosclerosis correlated to fewer podocyte, parietal, or tubular cell EVs, among others. Kidneys with nephron hypertrophy correlated to fewer mesangial or descending limbs of Henle’s loop cell EVs.	Turco A.E. et al. [58]
		LD vs. DD		miRNAs analysis, miR-326	No overall differences were found in EVs miRNA profiles of living and deceased donors in normofunctioning grafts at 1 year. Only miRNA-223, which targets pro-apoptotic protein Bcl-2, was found overexpressed in living donors.	Lozano-Ramos S.I. et al. [59]
Prediction of postoperative graft function	Kidney	Human TR with vs. without DFG	EVs in preservation fluid	Multiple miRNAs	Differences between the two groups were found in 10 miRNAs upon basic analysis, but differences were lost upon multiple testing correction. Groups were not successfully identified via unsupervised clustering in PCA.	Gremmels H. et al. [60]
		Human TR with vs. without DFG	Urinary EVs	Membrane protein (CD133+ EVs)	Patients with DFG had a significant increase in the CD133+ extracellular vesicle subpopulation compared with patients with early graft function. CD133+ may reflect the activity of progenitor cells in damage repair.	Dimuccio V. et al. [62]
		Human TR with vs. without DFG (from DD vs. LD)		NGAL	NGAL levels were higher in kidney recipients from DDs. NGAL levels are significantly higher in patients with DGF compared with early graft function.	Alvarez S. et al. [63]
		Human TR		NGAL, cystatin C, and IL-18 mRNA	Free urinary NGAL and cystatin C were correlated with serum creatinine at day 7 post-transplant. However, a poor correlation between EV-contained NGAL, cystatin C, or IL-18 mRNA and serum creatinine was found.	Peake P.W. et al. [64]
		Human TR		Proteomic analysis, phosphoenolpyruvate carboxykinase 2 (PCK2)	Proteomic profile 1-day post-transplant correlated with renal function at 1 year. PCK2 1-day post-transplant in uEVs, but not in renal tissue, could predict renal function at 1 year.	Braun F. et al. [119]
		Human TR with vs. without DFG (from DD)	Plasma-derived EVs	hsa-miR-33a-5p, hsa-miR-98-5p, and hsa-miR-151a-5p	A total of 52 miRNAs were found to be overexpressed in DGF compared with early graft function; of them, the mentioned 3 miRNAs were coexpressed. hsa-miR-151a-5p was positively correlated with first-week markers of graft function.	Wang J. et al. [65]
		Human TR with good vs. poor outcome based on eGFR		Proteomic analysis	Proteomic profile could differentiate patients with good outcomes from those with poor outcomes based on eGFR at 1 month.	Al-Nedawi K. et al. [66]
		Human TR with vs. without DFG (eGFR < 60 mL/min/1.73 m^2^) vs. healthy controls		miRNA analysis (miR-21-5p, miR-210-3p, and miR-4639-5p)	The panel could accurately differentiate between subjects with chronic allograft dysfunction and normal graft function, with better performance than simple or double indicators (ROC-AUC 0.89).	Chen Y. et al. [67]
Diagnosis of acute graft rejection	Kidney	Human TR with vs. without acute rejection	Urinary EVs	mRNA analysis (CXCL11, STAT1, SERPINA1, BMP7, NAMPT, IFNGR1, and IL18BP, among others)	This panel outperforms eGRF in early diagnosis of acute graft rejection (ROC-AUC 0.93). Moreover, it can differentiate mechanism of rejection (TCMR vs. ABMR).	El Fekih R. et al. [80]
		Human TR with vs. without ABMR vs. TCMR		Proteomic analysis, cystatin C (CST3), and lipopolysaccharide-binding protein (LBP)	The combination of EV-contained CST3 and LPS can accurately identify ABMR patients versus non-rejection patients (ROC-AUC 0.879 and 0.901, respectively), as well as to differentiate them from TCMR.	Kim M. et al. [77]
		Human TR with vs. without TCMR		Proteomic analysis Tetraspanin 1 (TSPAN1) and hemopexin (HPX)	TSPAN1 and HPX were significantly overexpressed in TCMR patients.	Lim J.H. et al. [78]
		Human TR with vs. without TCMR	T-cell-derived urinary EVs	Membrane protein (CD3)	Presence of T-cell-specific membrane marker CD3 could accurately predict TCMR (ROC-AUC 0.911).	Park J. et al. [81]
		Human TR with vs. without AR	Whole urine urinary EVs	Proteomic analysis	Eleven proteins were overexpressed in AR, three of which (CLCA1, PROS1, and KIAA053) were specific to the EV fraction.	Sigdel T.K. et al. [79]
		Human TR with vs. without ABMR	Plasma-derived EVs	Membrane markers (C4d+, CD144+, and annexin V+)	C4d+/CD144+ and C4d+/annexin V+ EV subpopulations were significantly increased in AR patients. Upon treatment, C4d+/CD144+ EVs significantly decreased.	Tower C.M. et al. [82]
		Human TR with vs. without ABMR vs. TCMR		mRNA analysis	Six genes were overexpressed in ABMR patients. A combination of 4 genes (gp130, SH2D1B, TNFα, and CCL4) can accurately predict ABMR.	Zhang H. et al. [83]
	Liver	Human TR with vs. without TCMR	Plasma-derived EVs	Multiple miRNAs	Expression of miRNAs was significantly different between AR and non-AR patients. miR-223 and let-7e-5p were up-regulated in AR patients, whereas miR-199a-3p was down-regulated.	Wang W. et al. [120]
		Human TR with vs. without TCMR	Plasma-derived EVs	Galectin-9	Levels of galectin-9 were higher in patients with acute TCMR.	Zhang A. et al. [87]
	Lung	Human TR with vs. without AR or BOS	Plasma-derived EVs BALF-derived EVs	Donor HLA; lung-associated self-antigens (collagen V [Col-V] and K alpha 1 tubulin [Kα1T]) miRNA analysis	EV-contained donor HLA and collagen V were significantly overexpressed in AR and BOS compared with healthy patients (*p* < 0.05). Collagen V was detected 3 months before AR and 6 months before BOS diagnosis. Differentially expressed immunoregulatory miRNAs were found for AR (miR-92a and miR-182) and BOL (previous ones and miR-142-5p and miR-155) compared with control.	Gunasekaran M. et al. [100]
		Human TR with vs. without AR	BALF-derived EVs	RNA analysis	Transcriptomic signatures were significantly different between patients with and without AR. Patients with AR showed overexpression of antigen-processing immune activation pathways.	Gregson A. et al. [99]
	Heart	Mice TR with vs. without AR	Plasma-derived EVs	Total plasma EV concentration	Total plasma EV concentration remained stable in control group, while it significantly decreased in the AR group at grade 0R rejection on histology. The model proved accurate for early prediction of AR (ROC-AUC 0.934) before any histology changes are detected.	Habertheuer A. et al. [93]
		Human TR with vs. without ABMR vs. TCMR		Membrane proteins (37 proteins)	AR EVs had increased concentration and decreased diameter. AR overexpressed HLA-I, CD41b, ROR-1, and SSEA-4 compared with controls. TCMR overexpressed CD2 and CD3, while ABMR overexpressed HLA-II, CD-326, CD19, CD20, and CD-25. The diagnostic model built on these markers reached a high accuracy (ROC-AUC 0.865)	Castellani C. et al. [94]
		Human TR with vs. without ABMR vs. TCMR		Proteomic analysis	A total of 45 EV-derived proteins were identified to differentiate 3 groups: control/heart failure group, heart transplant without rejection and, ABMR and TCMR. A total of 15 of them were differentially expressed between the 2 last groups (*p* < 0.05). Most of these proteins play a role in the immune response (complement activation, adaptive immunity, and coagulation).	Kennel P. et al. [95]
	Pancreatic islets	Mice TR from human islets, with and without induced AR	Plasma-derived EVs from donor’s islets and recipient T cells	EVs concentration, proteomic analysis, and miRNA analysis	AR led to a decrease in donor EVs and an increase in T cell recipient EVs. Four proteins were differentially expressed in AR versus control: angiopoietin 1, HSC70, C3, and hemopexin. Changes in microRNA and proteomic profiles were detected in AR prior to clinical effects (hyperglycemia).	Vallabhajosyula P. et al. [89]
Diagnosis of chronic graft rejection	Kidney	Human TR with chronic ABMR vs. healthy and other-cause damage (calcineurin inhibitors toxicity and interstitial fibrosis)	Urinary EVs	SYT17	Chronic ABMR patients had significantly higher SYT17 than the other groups. SYT17 could predict chronic ABMR with higher accuracy than traditional laboratory parameters (ROC-AUC 0.82).	Takada Y. et al. [84]
		Human TR with vs. without chronic ABMR		Proteomic analysis, APOA1, TTR, PIGR, HPX, AZGP1, and CP	Expression of the six proteins was increased in chronic rejection compared with long term graft survival.	Jung H.Y. et al. [85]
		Human TR with chronic allograft disfunction, with vs. without ABMR	T helper cells and plasma-derived EVs	Membrane proteins (CD4, CXCR5, CXCR3, and CTLA4)	The CD4+ CXCR5+ CXCR3- EV subpopulation was higher in ABMR patients, while expression of CTLA-4 was lower in this group.	Yang J. et al. [86]
	Lung	Human TR with vs. without BOS Mice immunized with EVs from patients with vs. without BOS	Plasma-derived EVs	Kα1T; Col-V MHC-II; costimulatory molecules, CD40, CD80, and CD86; and transcription factors (NF-κB, hypoxia-inducible factor 1-α, and IL-1R–associated kinase 1, among others)	The aforementioned proteins were overexpressed in BOS versus control patients. Mice treated with EVs from BOS patients developed a specific proinflammatory phenotype.	Gunasekaran M. et al. [121]
Diagnosis of ischemia reperfusion injury	Kidney	Mice with vs. without IRI Mice with vs. without IRI	Plasma-derived renal EVs	miRNA-23a	IRI increased miRNA-23a, which plays a role in macrophage activation. Inhibition of miRNA-23a ameliorated inflammation in the renal parenchyma.	Li Z. et al. [106]
				miR-374b-5p	Levels of miR-374b-5p were increased after IRI. Inhibition of miR-374b-5p would alleviate kidney injury, showing its role in the damage cascade.	Ding C. et al. [107]
		Rats with vs. without unilateral IRI	Urinary EVs	Aquaporin 1 (AQP1); fetuin-A	Glycosylated AQP1 secretion was significantly reduced in the first 6 h after IRI compared with controls or other causes of renal injury. AQP1 was also reduced in a TR patient 48 h after transplantation.	Sonoda H. et al. [104]
Diagnosis of infection	Kidney	Human TR with vs. without BK virus nephropathy	Urinary EVs	bkv-miR-B1-5p; bkv-miR-B1-5p/miR-16	Levels of viral miRNA (bkv-miR-B1-5p and bkv-miR-B1-5p/miR-16) showed a significant correlation with urinary BK viral load, as well as to plasma BK viral load, and could accurately predict viruria (ROC-AUC 0.989 and 0.985, respectively).	Kim M. et al. [116]
	Lung	Human TR with vs. without symptomatic respiratory tract infection Mice as recipients for EVs treatment	Plasma-derived renal EVs	Lung-associated self-antigens (collagen V [Col-V], K alpha 1 tubulin [Kα1T]), 20S proteasome, and viral antigens	EV-contained self-antigens and viral antigens were higher in recipients of symptomatic respiratory viral infections. Mice immunized with those EVs developed immune responses to self-antigens, such as fibrosis, small airway occlusion, and cellular infiltration.	Gunasekaran M. et al. [118]
Immunosuppressive drug monitoring	Kidney	Human TR under calcineurin inhibitor treatment with vs. without chronic calcineurin inhibitor toxicity vs. interstitial fibrosis and tubular damage from other causes	Urinary EVs	Proteomic analysis, CTSZ, RAB8A and SERPINC1	Members of the uroplakin and plakin families were significantly overexpressed in the group with calcineurin inhibitor toxicity. CTSZ, RAB8A, and SERPINC1 were significantly overexpressed in patients with toxicity compared with normally functioning ones.	Carreras-Planella L. et al. [109]
		Human TR under various immunosuppressive therapies and tacrolimus therapy		miRNA analysis	Expression of miR-155-5p and miR-223-3p showed significant correlation with tacrolimus dose and could be used to monitor toxicity. miR-223-3p also correlated with serum creatinine.	Costa de Freitas R. et al. [110]

All transplant recipients received allogenic grafts. All changes in the “reported outcomes” column were measured in EVs from the aforementioned origins. ABMR: antibody-mediated rejection, AR: acute rejection, BALF: bronchoalveolar lavage fluid, BOS: bronchiolitis obliterans syndrome, DD: deceased donor, DGF: delayed graft function, eGFR: estimated glomerular filtration rate, IRI: ischemia-reperfusion injury, NGAL: neutrophil gelatinase-associated lipocalin, LD: living donor, PCA: principal component analysis, TR: transplant recipient, and TCMR: T-cell-mediated rejection.

## Data Availability

No original data included.
